# CRISPR-Cas9 knockout screen identifies novel treatment targets in childhood high-grade glioma

**DOI:** 10.1186/s13148-023-01498-6

**Published:** 2023-05-09

**Authors:** Anna Wenger, Ida Karlsson, Teresia Kling, Helena Carén

**Affiliations:** Sahlgrenska Center for Cancer Research, Department of Medical Biochemistry and Cell Biology, Institute of Biomedicine, Sahlgrenska Academy, University of Gothenburg, Medicinaregatan 1F, 405 30 Gothenburg, Sweden

**Keywords:** Paediatric-type diffuse high-grade glioma, CRISPR-Cas9, Knockout, Cancer stem cell, *UBE2N*, *CHD4*, *LSM11*, *KANSL1*, *KANSL3*, *EED*

## Abstract

**Background:**

Brain tumours are the leading cause of cancer-related death in children, and there is no effective treatment. A growing body of evidence points to deregulated epigenetics as a tumour driver, particularly in paediatric cancers as they have relatively few genomic alterations, and key driver mutations have been identified in histone 3 (H3). Cancer stem cells (CSC) are implicated in tumour development, relapse and therapy resistance and thus particularly important to target. We therefore aimed to identify novel epigenetic treatment targets in CSC derived from H3-mutated high-grade glioma (HGG) through a CRISPR-Cas9 knockout screen.

**Results:**

The knockout screen identified more than 100 novel genes essential for the growth of CSC derived from paediatric HGG with H3K27M mutation. We successfully validated 12 of the 13 selected hits by individual knockout in the same two CSC lines, and for the top six hits we included two additional CSC lines derived from H3 wild-type paediatric HGG. Knockout of these genes led to a significant decrease in CSC growth, and altered stem cell and differentiation markers.

**Conclusions:**

The screen robustly identified essential genes known in the literature, but also many novel genes essential for CSC growth in paediatric HGG. Six of the novel genes (*UBE2N, CHD4*, *LSM11, KANSL1, KANSL3* and *EED)* were validated individually thus demonstrating their importance for CSC growth in H3-mutated and wild-type HGG. These genes should be further studied and evaluated as novel treatment targets in paediatric HGG.

**Supplementary Information:**

The online version contains supplementary material available at 10.1186/s13148-023-01498-6.

## Background

Cancer is the second most common cause of mortality in children in developed countries [1], and the leading cancer type is brain tumours [2]. Paediatric-type diffuse high-grade gliomas [3] (HGG; previously referred to as the separate entities glioblastoma or diffuse intrinsic pontine glioma), are one of the most aggressive brain tumours with a 5-year survival of only 18% [4]. Epigenetic mechanisms are crucial during embryogenesis and normal development, and aberrant epigenetic regulation has been suggested as a driver of tumourigenesis, especially in childhood cancer [5–7]. For example, childhood tumours have much fewer mutations and copy-number alterations compared to their adult counterparts [8, 9], but the existing mutations are frequently located in epigenetic components and modifiers (e.g. *H3F3A*, *DAXX* and *ATRX* for paediatric HGG) [10]. Mutations in histone 3 (H3) are in fact a key driver in paediatric-type HGG, where it is present in more than 70% of the tumours [10]. The mutations were initially considered exclusive to childhood tumours, but have since been found in a few adult tumours as well [11]. The WHO 2021 classification of paediatric-type diffuse HGG stratifies the tumours based on the mutation status of H3 (K27 altered, G34-mutant or wild-type) and location in the brain (hemispheric and midline) [3]. The H3 mutations take place on the histone tail, resulting in widespread alterations in gene expression and methylation pattern [12, 13]. DNA methylation pattern is increasingly used for classification and subtyping of all kinds of paediatric brain tumours, and prognostic methylation biomarkers have also been suggested [12, 14–16]. The distinct differences between adult and childhood cancer highlight the importance of studies using paediatric model systems. Our well-characterised primary cancer stem cell (CSC) lines are derived from paediatric HGG, with and without H3 K27 mutation [17], which allow for functional studies on the cells implicated in tumourigenesis, tumour relapse and treatment failure [18–21].

The Nobel Prize winning method CRISPR (clustered regularly interspaced short palindromic repeats) provides unprecedented ease of alterations in the genome [22–24]. Multiple CRISPR knockout screens have been performed, mainly in traditional cancer cell lines, to identify genes essential for cancer growth [25, 26]. We therefore performed a CRISPR knockout screen with an epigenetic/chromatin modifier library (including, e.g. chromatin-remodelling genes and histone modifiers) on two CSC lines derived from H3 K27-mutated paediatric HGG. We identified novel genes as essential for CSC growth, with an enrichment for histone acetyltransferase and methyltransferase complexes. We validated our findings by individual knockout in the two H3-mutated CSC lines and extended with two H3 wild-type CSC, to examine on-target and potential off-target effects, stemness and differentiation markers and inhibitors. The tumour specificity of the gene knockout was tested in neural foetal stem cells (NSC), and our results show that the genes *UBE2N*, *CHD4*, *LSM11, EED, KANSL1* and *KANSL3* are essential for CSC growth and suggest them as therapeutic candidates in paediatric HGG.

## Results

### CRISPR knockout screen robustly identifies essential genes

**Fig. 1:**
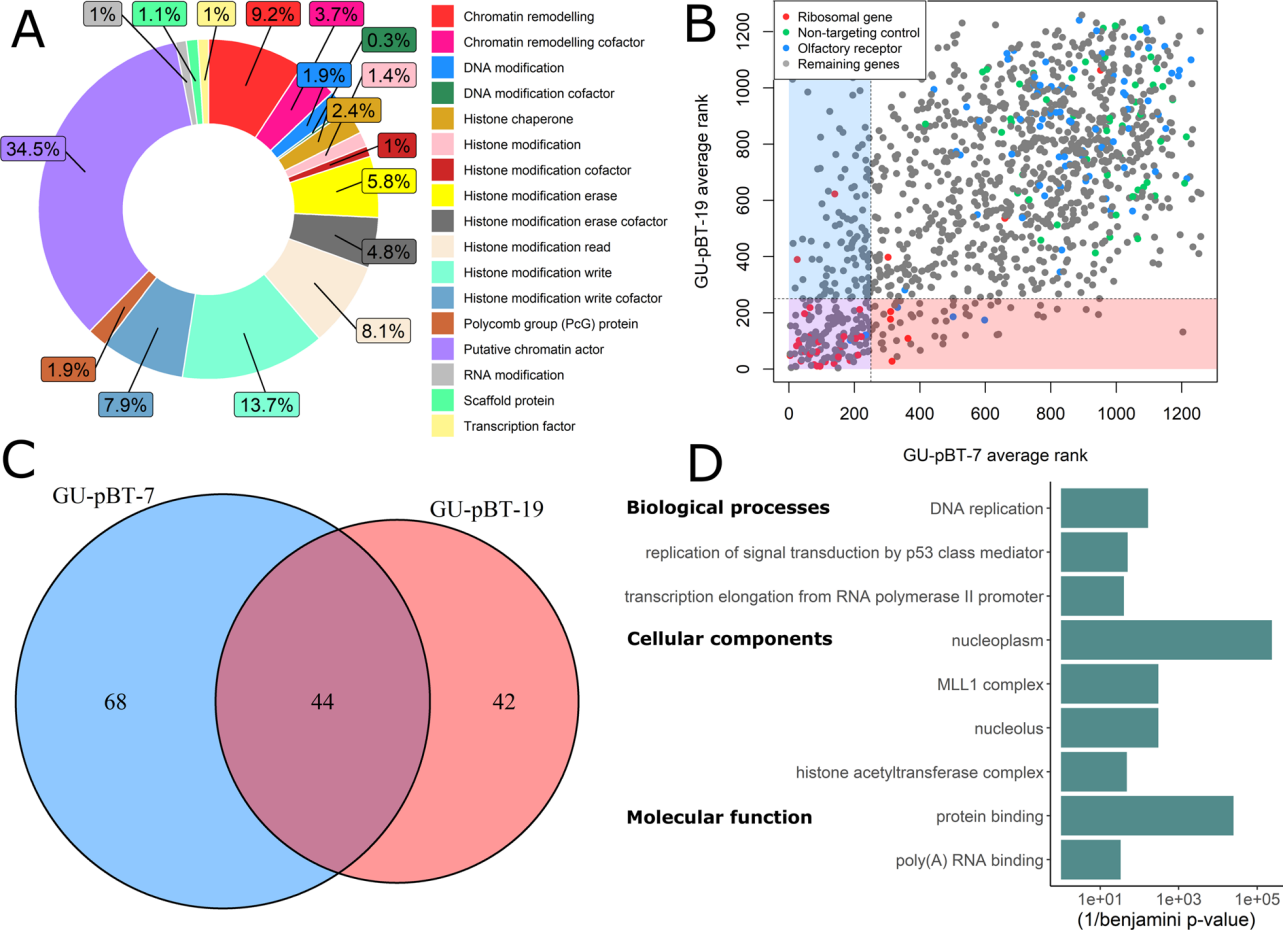
Screen library and hit calling. **A** Epifactor classification of the genes in the screen. **B** Average rank (of technical duplicates) for GU-pBT-7 vs GU-pBT-19 in the knockout screen. Lower rank indicates more essential/depleted after knockout. We set a threshold at ranking ≤ 250 for hit calling, with at least 3 (out of 4) concordant gRNA and replicate overlap. **C** These criteria resulted in 154 hits, where 44 were shared between the cell lines. **D** The significantly enriched GO terms for the hits (compared to the screen background) are presented

Two CSC lines derived from H3 K27-mutated paediatric HGG were used for a CRISPR knockout screen with an epigenetic/chromatin modifier library [27] (Fig. 1A, Additional file 1: Fig. S1A, Additional file 2: Table S1). The screen data were analysed with the rank-based MaGeCK software [28], where a lower rank indicates a better hit and cell depletion after knockout. We aimed to identify the genes that upon knockout led to cell death or reduced proliferation for the CSC (Additional file 1: Fig. S1B). The log fold change (LFC) distribution and correlation between the replicates in the knockout screen was in good agreement (*r* = 0.7 and 0.6, respectively, for GU-pBT-7 and GU-pBT-19; Additional file 1: Fig. S1C–E; Additional file 3: Table S2). The MaGeCK ranking results showed that the screen robustly identified the positive controls included in the screen, whereas the negative controls had high ranking scores (Fig. 1B). Based on the ranking of the positive controls, we set a threshold for hit calling at the top 250 for both replicates with at least three (out of four) concordant gRNA and potential false-positive hits were filtered away. This strategy identified 154 hits in the CSC; 44 shared hits between the cell lines, and 68 and 42 individual hits, respectively (Fig. 1C). Significantly enriched GO terms for the hits included histone acetyltransferase and methyltransferase (MLL1) complexes, DNA replication and protein binding (Fig. 1D). This was also supported by the Epifactors Database classification of the hits, where genes classified as histone chaperones, histone write factors and histone write cofactors were overrepresented compared to the screen background (Additional file 1: Fig. S1F).

### Selection of novel hits essential for CSC

**Fig. 2:**
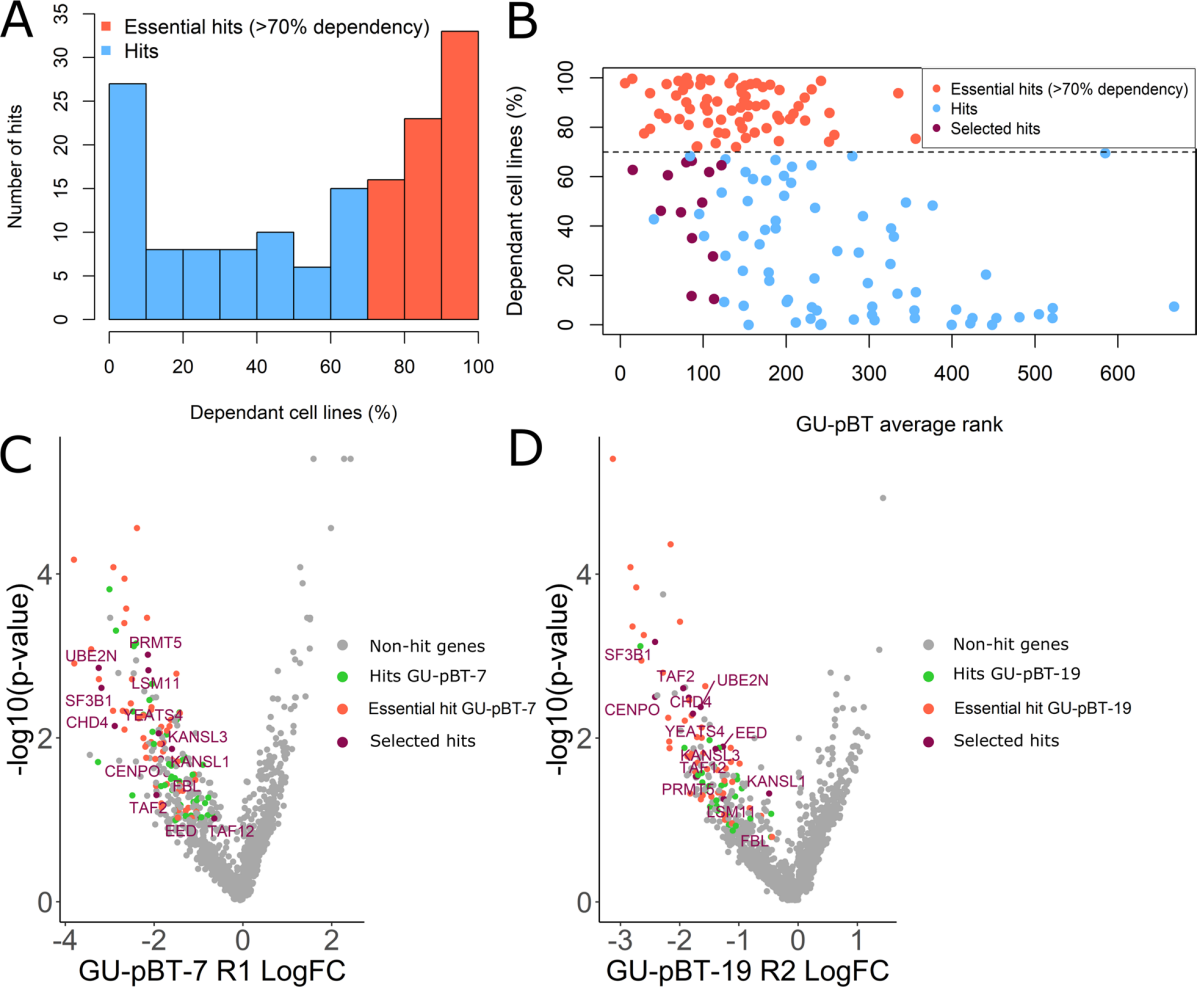
Selection of novel hits sparing for non-cancer cells. **A** We used data from knockout screens in > 300 traditional cancer cell lines listing for each gene how many of the cell lines (in percentage) that were dependent on the gene. The histogram shows the dependency distribution of all hits in our screen where hits with > 70% dependency are coloured in red. **B** The average rank of GU-pBT-7 and GU-pBT-19 (with technical duplicates for each cell line) vs dependent cell lines (according to Behan et al.) for all 154 hit genes in the screen. The dashed line indicates 70% dependency, which was used as a threshold for selecting 13 hits for individual validation (shown in purple). The hit genes above 70% dependency are coloured in red, and the remaining hits in blue. **C** Volcano plot of knockout screen results for GU-pBT-7 replicate 1 and **D** GU-pBT-19 replicate 2, of all genes in the screen where the 13 selected hits are labelled in purple. Hit genes above 70% dependency are coloured in red, remaining hit genes for each cell line is shown in green, and the remaining genes (non-hits) in the screen in grey

The hit list in our screen reassuringly contained many well-known cancer genes such as *PCNA*, *CDK1* and *PLK1,* further demonstrating the validity of the screen results. However, these genes have been termed as essential for all cells and studied extensively [25, 26, 29], and for further investigations in this study we selected genes that are fairly novel in HGG and potentially sparing for normal cells. To filter out common essential hits, we excluded those genes that were a hit in more than 70% of the screened 324 cancer cell lines according to Behan et al. [25] (Fig. 2A). This excluded 72 of our 154 hits (47%). We then selected 13 of the remaining best-ranking hits in the CSC (Fig. 2B–D; Additional file 1: Fig. S2) for validation and mechanistic investigations. Nine of these 13 were hits in both cell lines and four were hits in one of the cell lines and very close to a hit in the other. The selected genes included, e.g. *CHD4*, a chromatin-remodelling factor that is part of the NuRD (nucleosome remodelling and deacetylase) complex, *EED*, part of the polycomb repressive complex 2 (PRC2) complex, and *UBE2N* (also known as Ubc13), an ubiquitin-conjugating enzyme that promotes the loss of tumour suppressor p53 [30].

### Optimisation for individual knockout

**Fig. 3:**
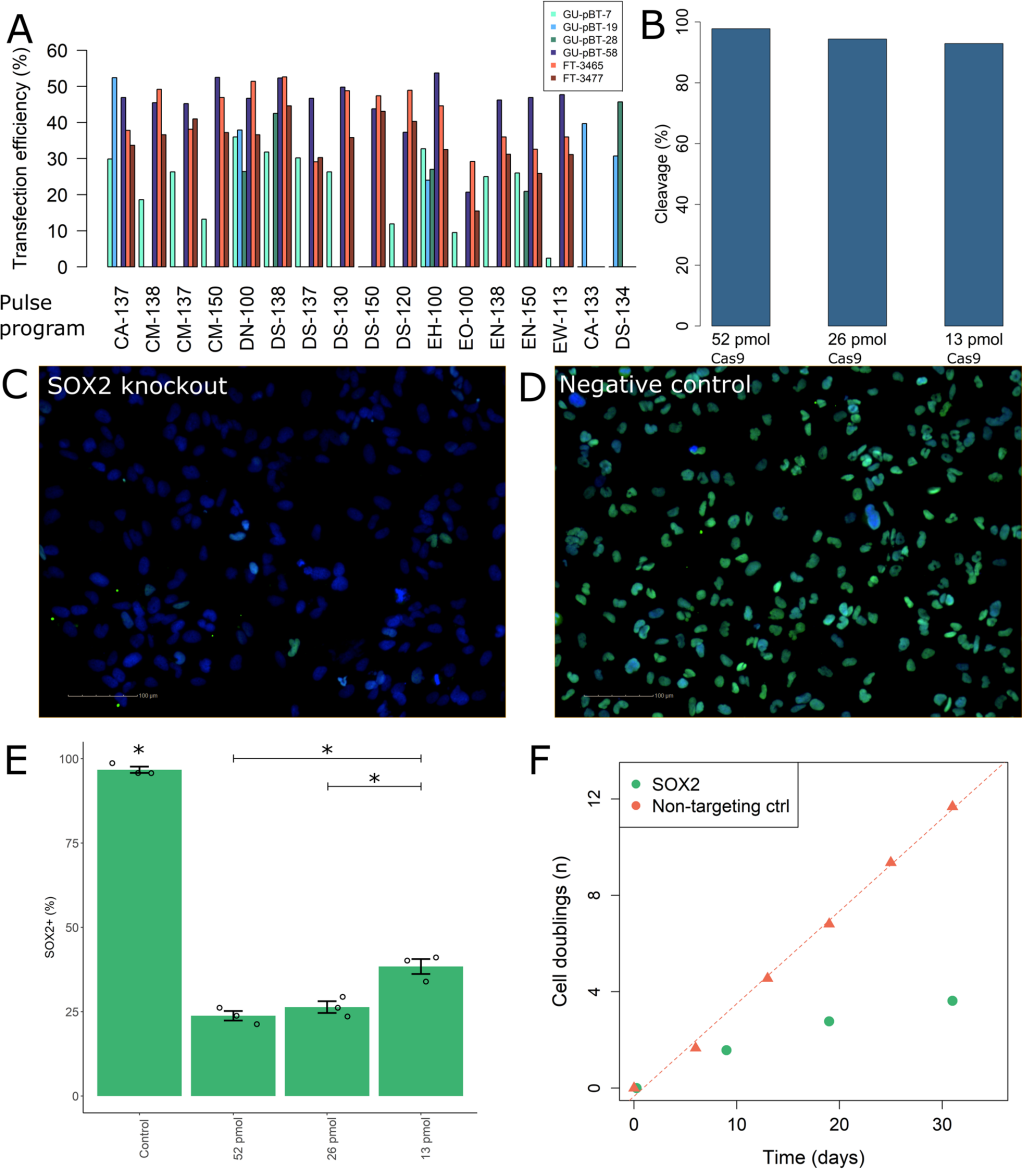
Optimisation of individual knockout. **A** All cell lines were nucleofected with a GFP plasmid using multiple pulse programs. The program that yielded the best transfection efficiency (number of GFP + cells divided with number of live cells in the control) and morphology was selected for each cell line (DN-100 for GU-pBT-7 and FT3465, CA-133 for GU-pBT-19, DS-134 for GU-pBT-28, CM-150 for GU-pBT-58, DS-150 for FT3477). **B** SOX2 was knocked out in GU-pBT-7 through nucleofection of RNP with different amounts of Cas9 (constant gRNA-to-Cas9 ratio) and the DNA cleavage decreased with decreasing amounts. **C** SOX2 knockout demonstrates loss of protein (green nuclei indicate SOX2 staining; DAPI in blue as counterstain for all nuclei) compared to **D** the control, and **E** more cells lose the SOX2 protein with increasing amounts. The control has significantly (*p* value < 0.05; Welch one-sided t test; indicated by *; n = 3 technical replicates for all conditions) higher proportion than all knockout condition. Knockout with 13 pmol Cas9 has significantly higher proportion of cells with retained SOX2 compared to 26 and 52 pmol. The error bars indicate standard error mean. **F** The SOX2 knockout cells and control cells were cultured for one month and counted at each split. The SOX2 knockout grew significantly slower than the control (*p* value < 0.05; Welch one-sided t test)

In order to validate the hits from the screen and study the phenotype after knockout, we set up a method for individual knockout. We selected nucleofection of RNP complexes of the Cas9 and gRNA (duplex of crRNA/tracrRNA) instead of lentivirus, since RNPs are highly efficient, yield faster cleavage as the cell does not need to produce the components and reduce off-target effects [31, 32]. First, individual pulse programs were selected for the cell lines based on viability, number of GFP + cells and morphology 24 h post-nucleofection (Fig. 3A). Next, the transcription factor *SOX2* (*sex determining region Y – box 2*), a known stem cell marker required for self-renewal of glial stem cells [33], was knocked out. We tested different concentrations of RNP (while maintaining the Cas9-to-gRNA ratio) to minimise the RNP amount needed to achieve a high cleavage efficiency and loss of protein. The uptake of the RNP complex in the cells was verified visually by the fluorescent ATTO tracrRNA (Additional file 1: Fig. S3A), and ICE (Inference of CRISPR Edits) [34] analysis showed > 90% cleavage efficiency at the intended target with a significant loss of protein in 73% of the cells 48 h after knockout (Fig. 3B–E; Additional file 1: Fig. S3B). Finally, the cells were cultured for one month after knockout and counted at each split and, as expected, the cells knocked for *SOX2* vastly decreased their proliferation (Fig. 3F). This pipeline for RNP knockout by nucleofection can thus be used in our primary patient-derived CSC to validate the hits from the screen.

### Selected hits significantly reduce cancer stem cell growth upon knockout

**Fig. 4:**
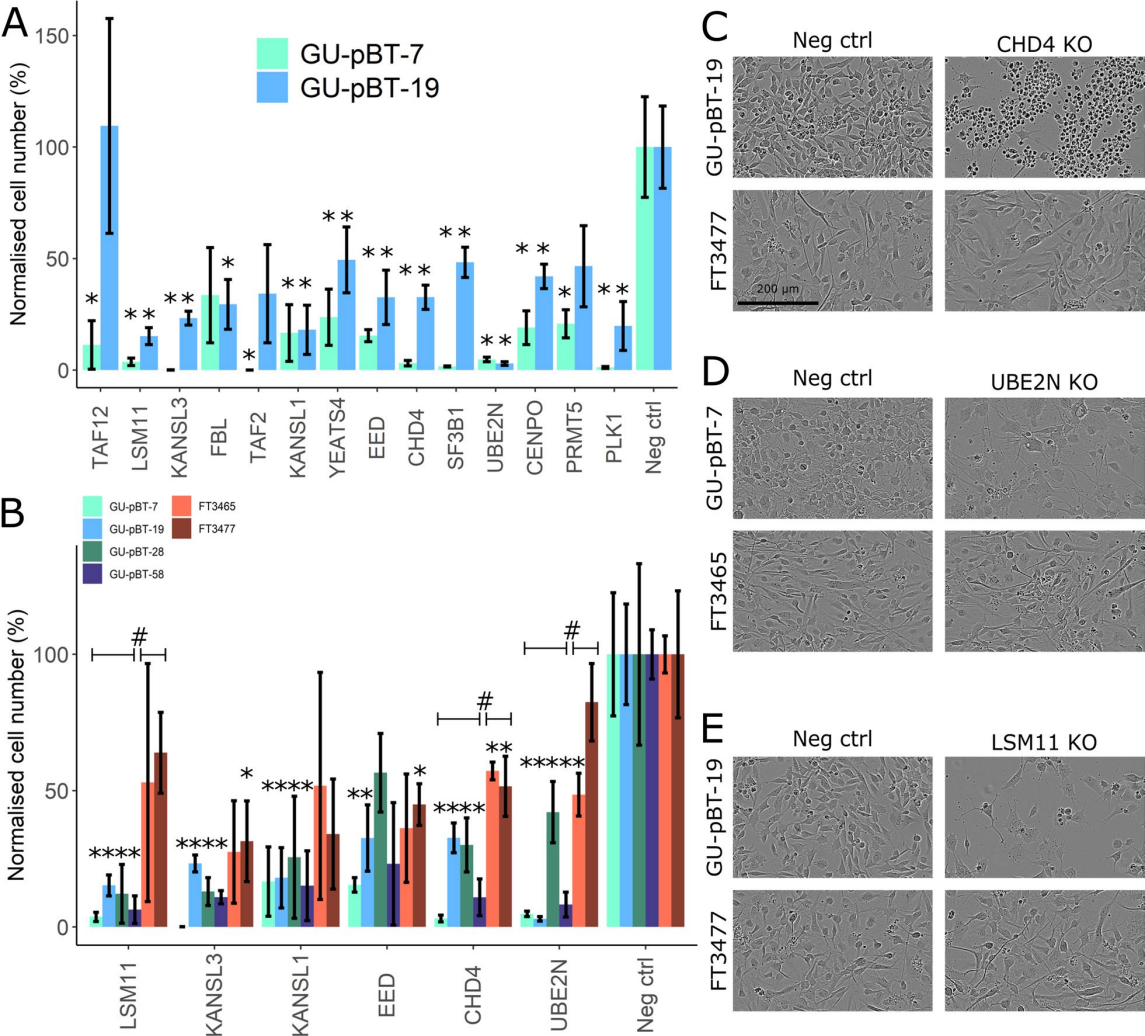
Successful validation in CSC. **A** Thirteen genes were individually knocked in GU-pBT-7 and GU-pBT-19 (n = 2 gRNA per gene, i.e. biological replicates, except negative control where n = 3 or 4 samples) and cultured for one month. The cell counts at each split was summarised and used to compare the growth of the knockout cells to the negative controls. All genes significantly decreased (Welch t test, *p* value < 0.05; indicated with * above the bar) the CSC growth after knockout compared to the control for at least one of the cell lines. PLK1 was included as a positive control. **B** The six genes that showed the most effect were selected for further validation in two additional CSC lines and tested in two NSC lines (n = 2 gRNA per gene). Note that the data for GU-pBT-7 and GU-pBT-19 are from the same experiment as presented in the A-panel. Error bars represent the standard error mean. * above a bar indicates significant decrease in cell number for that gene knockout compared to the negative control for the cell line in question. # (with associated braces indicating the CSC and NSC lines) denotes a significant difference at group level between the CSC and NSC in the case of UBE2N, LSM11 and CHD4 knockout, respectively (Welch t test, *p* value < 0.05). **C** CHD4 knocked out in the CSC GU-pBT-19 showed drastic reduction in cell viability, but the NSC FT3477 were not affected morphologically, which was also seen for knockout of **D** UBE2N and **E** LSM11. The cell images are from around 10 days after knockout. Scale bar = 200 µm applies to all images in the C-E panel

The optimised pipeline was used to individually knockout the 13 selected hits with RNP in the same cell lines used in the screen (GU-pBT-7 and GU-pBT-19; both H3 K27 mutated). The total cell number during the experiment period (one month) was normalised against the negative controls (knockout of olfactory receptor, non-targeting control and a non-nucleofected sample; see methods for details). Twelve of the 13 genes (92%) were successfully validated, as knockout of these genes elicited a large decrease in cell number in both cell lines (significant decrease in both cell lines for 10/13 genes), thus validating the genes’ importance for CSC growth in H3K27M HGG (Fig. 4A). The effect was seen immediately for some genes, such as *KANSL3,* whereas others, e.g. *UBE2N*, gradually decreased the cell growth (Additional file 1: Fig. S3C, D). The six genes (*UBE2N, CHD4, KANSL1, KANSL3, LSM11* and *EED)* that yielded the largest effect on cell number in both cell lines were selected for further evaluation. We knocked out the six genes in two additional CSC (both derived from H3 wild-type paediatric HGG) and also in two NSC lines. The gene knockouts had a significant effect on these CSC as well (except for *EED*), demonstrating a robust effect on cell proliferation/viability in a range of CSC lines regardless of the H3 mutation state (Fig. 4B). The effect varied between the two NSC and the cell growth was significantly reduced in one or both of the cell lines after *KANSL3*, *EED*, *CHD4* and *UBE2N* knockout. However, the *LSM11*, *CHD4* and *UBE2N* knockout had a significantly larger effect on CSC growth than the NSC. Gene knockout in NSC was also less detrimental in terms of morphology (Fig. 4C–E). For example, knockout of *CHD4* in CSC elicited massive cell death that was not observed in the NSC, even though their growth was affected, and the NSC had no apparent change in their morphology (Fig. 4C). In particular, knockout of *LSM11*, *CHD4* and *UBE2N* had the least effect on NSC growth (significant difference between CSC and NSC response) and morphology (Fig. 4B–E), suggesting that normal cells may tolerate the loss of these genes, whereas it is essential for the CSC.

### Knockout of the candidate genes alter stem cell and differentiation markers

**Fig. 5:**
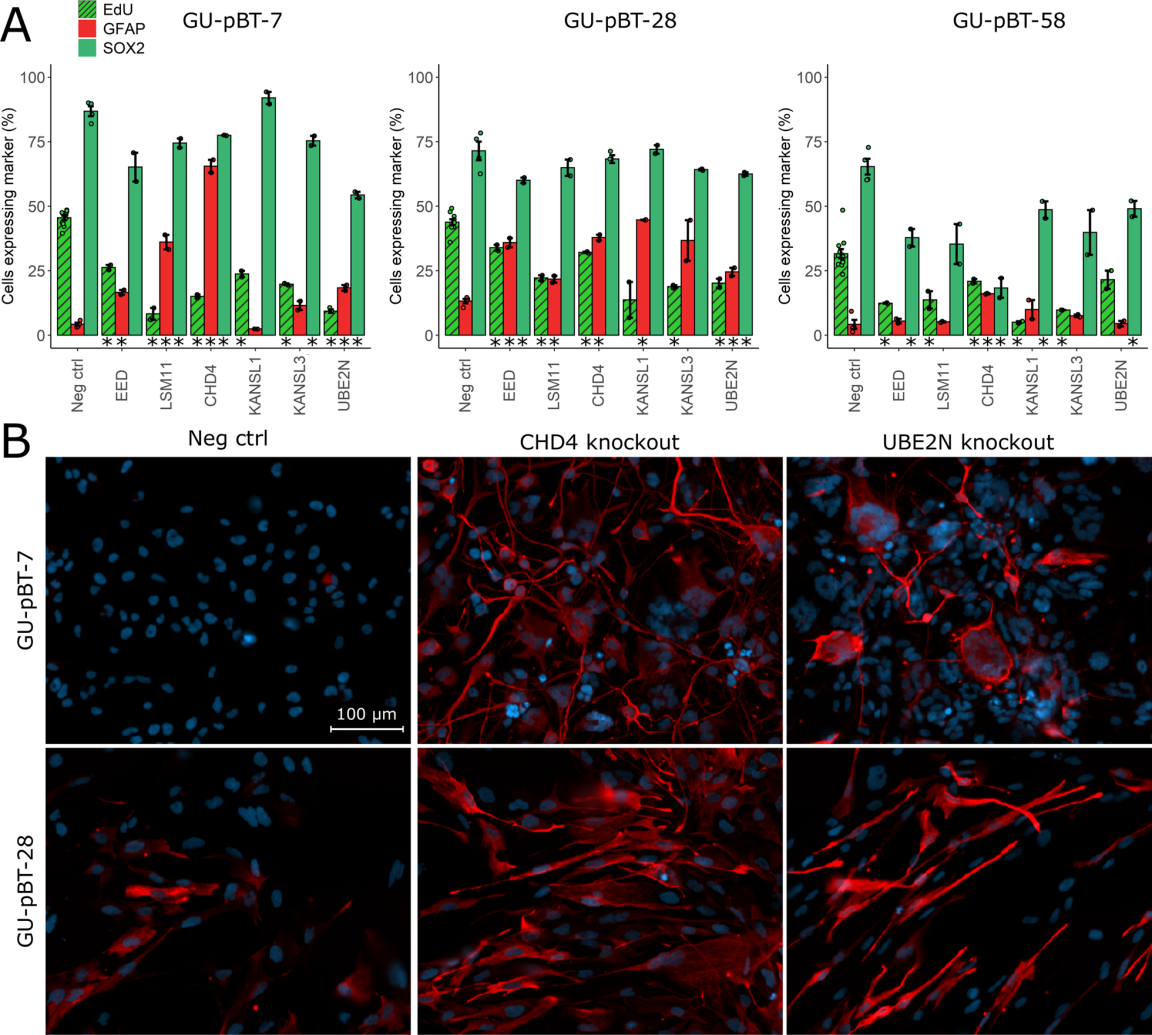
Knockout induces alterations in stem cell and differentiation markers. **A** Knockout of the candidate genes leads to decreased proliferation (EdU) and expression of stem cell marker SOX2, and increase in the differentiation marker GFAP. An asterisk (*) under a bar denotes significant difference of the marker compared to the negative control for the cell line (Welch one-sided t test, *p* value < 0.05). For example, knockout of EED in GU-pBT-7 leads to significantly decreased proliferation rate (EdU) and significantly increased expression of GFAP compared to the negative control of GU-pBT-7, while the decrease in SOX2 is not statistically significant (*p* value = 0.065). Error bars represent the standard error mean (n = 2 technical replicates per knockout condition and staining. EdU ctrl n = 12 technical replicates, GFAP and SOX2 ctrl n = 4 technical replicates). **B** A large increase in GFAP was in particular seen for CHD4 and UBE2N knockout, respectively. Scale bar = 100 µm applies to all images

On-target cleavage effect at the intended loci and potential off-target effects at two of the top putative genome locations were evaluated with ICE analysis approximately 10 days after knockout. The on-target cleavage efficiency was > 80%, and no off-target effects were observed (Additional file 1: Fig. S4). We also validated loss of protein after knockout using immunocytochemistry with antibodies against CHD4 and UBE2N (Additional file 1: Fig. S5A–C). Next, we found that knockout of several of the candidate genes led to significantly higher expression of the differentiation marker glial fibrillary acidic protein (GFAP), while the proliferation marker EdU and stem cell marker SOX2 decreased (Fig. 5A). The increase in GFAP was most noteworthy for CHD4 knockout (significant increase in all three tested cell lines) and UBE2N (Fig. 5B).

### Several hit genes are suitable for therapeutic intervention

**Fig. 6:**
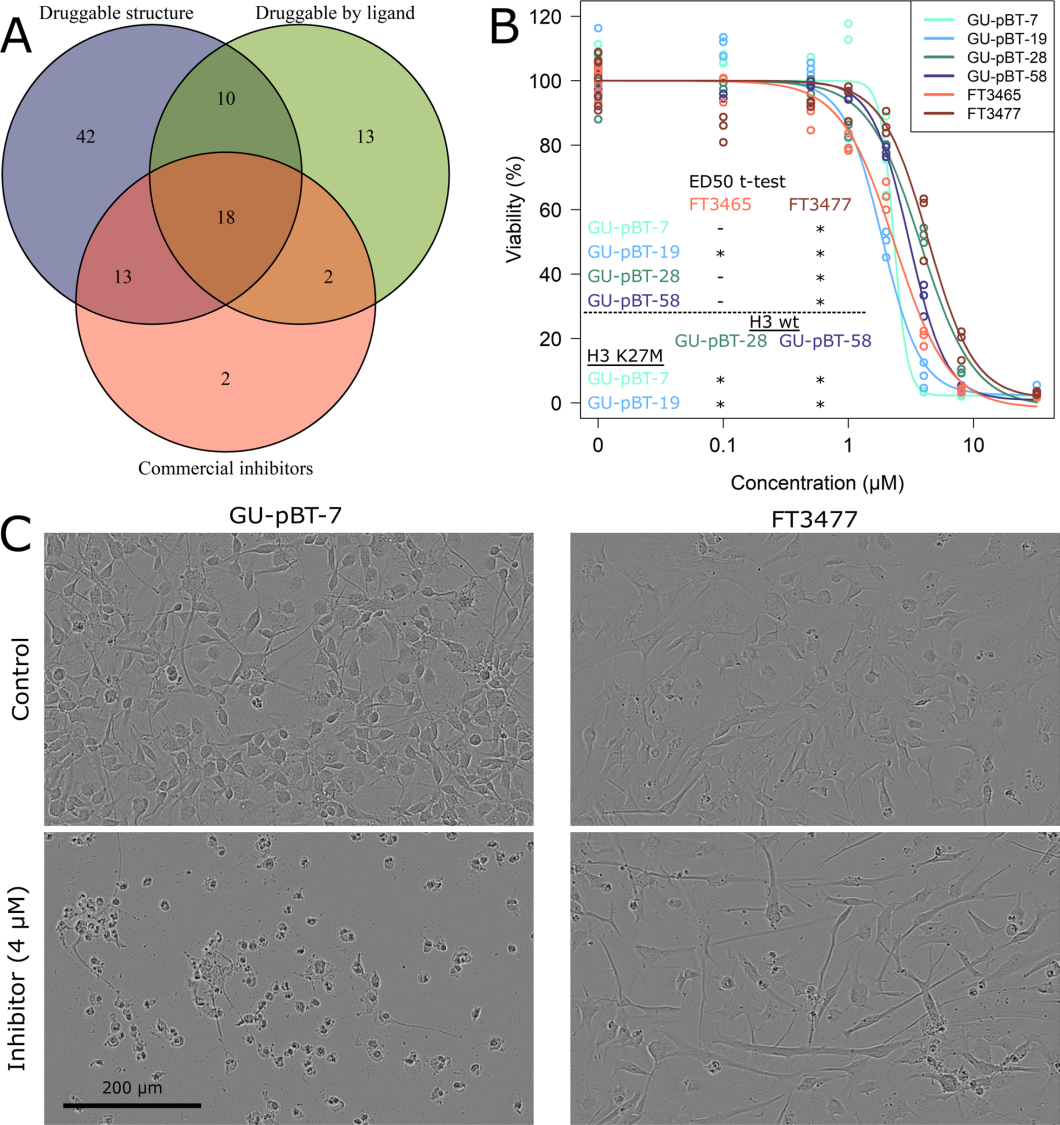
Therapeutic potential of hits. **A** The hit genes in the screen on GU-pBT-7 and GU-pBT-19 were examined with canSAR to predict their druggability. We also did a web-based search for the hits that had commercially available inhibitors. One of the candidates (UBE2N) had a commercial inhibitor (NSC697923), and a dose–response curve for **B** four CSC and two NSC lines showed that the inhibitor killed all cells at the highest concentration tested (32 µM; n = 3 technical replicates per condition except for the control where n = 12 technical replicates). The ED50 (median effective) was significantly higher in the NSC FT3477 compared to all CSC (* indicates *p* value < 0.05 in one-sided Welch t test of the ED50). The ED50 was also significantly higher in the H3 wild-type cell lines (GU-pBT-28 and GU-pBT-58) than the H3K27M cell lines (GU-pBT-7 and GU-pBT-19). **C** Phase images of the CSC GU-pBT-7 (left) and NSC FT3477 (right) with untreated cells (top) and treated with the inhibitor (4 µM) for 4 days. Scale bar = 200 µm

We were next interested in the therapeutic potential of the hit genes in our screen, i.e. can the proteins be inhibited pharmacologically rather than through gene knockout? 83 of the 154 hits were predicted to have druggable structures by canSAR [35] (Fig. 6A), a drug discovery database for oncology (https://cansarblack.icr.ac.uk/). Fifteen additional proteins were predicted as druggable by ligand-based assessment. A web-based search revealed that 35 of the hits had commercially available inhibitors. One of these was directed against the candidate UBE2N (NSC697923 inhibitor), and we therefore tested it on four CSC (two H3 wild-type and two K27 mutated) and two NSC lines. The UBE2N inhibitor promisingly had a large effect on all tested CSC lines, killing all cells at the highest examined concentrations (Fig. 6B). It is noteworthy that the two H3-mutated cell lines (GU-pBT-7 and GU-pBT-19) responded best to the inhibitor and their ED50 values were significantly lower (p value < 0.05) than GU-pBT-28 and GU-pBT-58 (both H3 wild-type), respectively. The inhibitor’s effect on the CSC was clearly visible already within one day after treatment start (Additional file 1: Fig. S6), suggesting a toxic effect. In comparison, the cells responded slower to the *UBE2N* knockout and gained a different morphology and slowed growth rate over time. The NSC were also killed by the inhibitor, but one of the cell lines (FT3477) tolerated it significantly better than all CSC (Fig. 6B–C), indicating a potential therapeutic window particularly for H3K27-mutated childhood HGG.

## Discussion

The earlier increase in survival has plateaued for childhood cancer during the last decades [1]. Further knowledge on the tumour pathogenesis is needed to identify new treatment targets and therapies, both to improve survival but also to decrease adverse effects. Paediatric brain tumour survivors frequently experience long-term side effects caused by harsh treatments (such as radiation therapy) to a brain that is still developing [36, 37]. Functional studies are needed to advance our understanding and identify essential genes in HGG. Given the molecular differences between paediatric and adult HGG [10, 38], it is vital that the model systems used are representative of the paediatric disease. We therefore performed an epigenetic knockout screen of 1200 genes in our patient-derived CSC from childhood H3K27-mutated HGG. Our screen robustly identified positive controls (ribosomal genes), previously identified essential core genes, and novel genes in the HGG setting. In total, we identified 154 hits in paediatric H3-mutated HGG. Not only are our cell lines representative of the patient tumours [17], but since they are CSC, the screen identified essential genes in the cells that are driving the tumour initiation and progression. The identified hits in the CSC were significantly enriched for GO terms including DNA replication, histone acetyltransferase and methyltransferase (MLL1) complex.

Several of the hits in our screen (such as *PLK1* and *CDK1*) have previously been labelled as “core essential”, “fitness genes” or “pan-cancer” genes [25, 26, 29], as they are considered essential for all cells (cancer and healthy cells) to function. That does not automatically imply that they are bad treatment targets as targeted delivery through, for example, lipid nanoparticles has been demonstrated as a feasible option [39]. These genes are also important from a biological standpoint to understand the disease pathogenesis. *PLK1* has previously been reported as essential in paediatric midline gliomas [40, 41], and our results validate those findings warranting further studies. PLK1 inhibitors exist, but concerns regarding their toxicity on normal cells have been raised [42]. We therefore decided here to focus on genes that are not core essential and thus set a threshold of less than 70% dependency [25] for the hits that were selected for validation. This criterion was fulfilled by 82 of our hits (53%), providing an abundant number of hits predicted as specific targets in childhood HGG compared to healthy tissue. Further, 38 of these 82 were predicted by canSAR [35] to have druggable structures, and an additional 11 to be amendable to ligand-based drugging.

Six of our hits were knocked out in NSC, and we noted, in all cases, trends that the NSC tolerated the knockout better than the CSC. The difference was significant for three of the genes (*LSM11*, *CHD4* and *UBE2N*). The findings suggest that these three genes are essential for CSC, and as such potential drivers of the tumour and represent novel treatment targets in HGG. We also tested the commercially available UBE2N inhibitor, NSC697923, and it efficiently killed all CSC, further verifying the importance of UBE2N in CSC and HGG. The median effective value of the inhibitor (ED50) was significantly lower in the two H3K27M cell lines (GU-pBT-7 and GU-pBT-19). The inhibitor seemed to induce a toxic response in CSC as well as NSC, even though one of the NSC lines was significantly more resistant to the inhibitor than the CSC, particularly the H3K27M-derived CSC. Such rapid decrease in cell growth was not observed in the cells after knockout of UBE2N, where instead a decrease in proliferation and SOX2 together with an increase in GFAP was observed, indicative of cell differentiation. These changes were also seen after knockout of the other candidate genes suggesting that loss of these candidate proteins may induce differentiation of the CSC and contributing to the slowed growth that we observed.

KANSL3 and LSM11 are novel targets as not much is previously published about them in regards to cancer. LSM11 is, however, believed to be involved in cell cycle regulation and cell growth, providing a potential explanation why loss of LSM11 led to decreased growth in CSC as observed here. KANSL3 and KANSL1 are subunits of the nonspecific lethal (NSL) 1 complex that regulates gene transcription through, for example, histone acetylation [43]. *KANSL1* has also been reported as frequently amplified or rearranged in ovarian cancer [44] and mutated in relapsed acute myeloid leukaemia (AML) [45]. *UBE2N* is associated with several cancer types including neuroblastoma, breast cancer and B cell lymphoma [46]. Interestingly, UBE2N promotes cytoplasmic translocation of p53 and subsequent loss of function of this vital tumour suppressor [30, 47]. Inhibition of UBE2N in neuroblastoma resulted in nuclear accumulation of p53 and restored function of the protein [48], and a similar mechanism may exist in HGG. A knockout screen on tumour sphere cells derived from diffuse intrinsic pontine glioma also ranked knockout of *UBE2N*, *CHD4* and *EED* as a hit in combination with the histone deacetylase inhibitor panobinostat [49]. *CHD4*, like *UBE2N*, is also involved in several cancer types and has been reported as essential for the maintenance of AML [50], and required for tumour cell survival in adult HGG [51]. *EED*, a core part of the PRC2, has in itself been associated with several forms of cancer, as has the PRC2 complex [52]. *EED* was also identified as a hit in a recent CRISPR knockout screen in tumour spheres derived from H3-mutated and wild-type childhood HGG [53].

The optimisation of our pipeline for individual knockout in paediatric patient-derived CSC was essential to routinely obtain high DNA cleavage efficiencies (> 80%), similar to previous publications using RNP [54, 55], and nucleofection efficiency in the same range as previously reported for NSC [56]. Regarding off-target effects, no DNA cleavage was detected at two of the top predicted sites in the genome. This was likely a result of the use of RNP, which are known to cause less off-target cuts compared to plasmids etcetera [31, 32].

## Conclusions

In conclusion, we have performed a CRISPR knockout screen on paediatric H3K27-mutated HGG with CSC lines accurately reflecting the disease. The screen robustly identified the positive controls and essential genes known in the literature, but also several novel genes essential for CSC growth in paediatric HGG. Interestingly, many of the hit genes were predicted as druggable highlighting their therapeutic potential, and we evaluated an UBE2N inhibitor, which successfully killed the CSC. We optimised an individual knockout pipeline (nucleofection of RNP complexes) for the cell lines routinely achieving high DNA cleavage efficiencies (> 80%) and loss of protein while detecting no off-target effects at the top predicted locations. The optimised pipeline was used to successfully validate 12 genes, demonstrating their importance for CSC growth. Six of these genes were knocked out in four CSC lines, derived from H3 wild-type as well as H3K27-mutated HGG, proving their broad effect on cell growth. The decrease in cell growth may partially be caused by differentiation as the expression of the astrocytic marker GFAP increased, while stem cell markers decreased, but this requires further studies. We also knocked out the six candidate genes in NSC, and while their viability was affected by the knockout, it was not as detrimental as for the CSC, especially for the genes *UBE2N*, *CHD4* and *LSM11*. Our results suggest that the six candidate genes *UBE2N, CHD4, LSM11, KANSL1, KANSL3* and *EED* are vital for CSC growth, and potential therapeutic targets in paediatric HGG.

## Methods

### Experimental study design and statistical analysis

No formal sample size estimation was performed as well-characterised cell lines from paediatric high-grade gliomas are very rare. We therefore used two CSC lines, which could tolerate the lentiviral transduction of the knockout library, for the CRISPR knockout screen. To compensate for the relatively small sample size, we used stringent thresholds for hit calling and used four CSC lines in the validation step. Blinding was not performed as, for example, specific pulse programs for nucleofection were required for the different cell lines.

P values for the pooled CRISPR knockout screen was calculated according to the MAGeCK software [28]. Validation by individual knockout was performed as described above, and the cell counts during one month after knockout were used to calculate the number of cell doublings between the splits according to:

$$n = \frac{{{\text{log}}\left( {\frac{{{\text{harvested }}\;{\text{cells}}}}{{{\text{seeded }}\;{\text{cells}}}}} \right)}}{{{\text{log}}\left( 2 \right)}}$$

The number of cell doublings during the one-month culture was summed up for each gRNA and used to calculate the total number of cells. An average of the two gRNA for each gene was calculated and then normalised to the total cell number for the negative controls (*n* = 3 or 4). Welch one-tailed t test was used (as samples were tested for and demonstrated unequal variance) to test if the cell growth after gene knockout was significantly different compared to the growth of the negative controls of the cell lines. It was also used to test if the growth after knockout was significantly reduced in CSC compared to NSC on a group level (Fig. 4B), and if the proportion of cells expressing a marker (SOX2, GFAP, EdU; Fig. 5A) was significantly altered in the knockout condition compared to the negative control. A *p* value < 0.05 was considered significant (indicated by * in the figures).

The dose–response curve (Fig. 6B) was fitted with a 3-parameter log-logistic model and the ED50 (median effective dose) using the R package drc (Additional file 4: Table S3) [57]. Welch one-tailed t test was used to test if the ED50 was lower in the individual CSC lines compared to each of the NSC lines. We also tested the if the ED50 was lower in the H3K27M CSC compared to the H3 wild-type CSC lines. A *p* value < 0.05 was considered significant.

### Cell culture

The study was approved by the regional ethics committee in Gothenburg (Dnr 604–12) and carried out according to relevant guidelines and regulations. The cell lines GU-pBT-7 (male, 4 years, diffuse midline glioma H3 K27 altered, thalamus location), GU-pBT-19 (male, 6 years, diffuse midline glioma H3 K27 altered, thalamus location) and GU-pBT-28 (female, 11 years, diffuse paediatric-type high-grade glioma H3 wild-type and IDH wild-type, pons location) have been characterised and presented previously [17, 58], and GU-pBT-58 (female, 3 years, diffuse paediatric-type high-grade glioma H3 wild-type and IDH wild-type, pons location) was isolated according to the same protocol. Informed consent was obtained from the parents of all patients and the experiments conformed to the principles set out in the WMA Declaration of Helsinki and the Department of Health and Human Services Belmont Report. STR profiling (Identicell, Aarhus, Denmark) verified the uniqueness of the cell lines, and the cells were negative for mycoplasma contamination (Eurofins Genomics, Ebersberg, Germany).

The foetal NSC were a kind gift from Professor Steven Pollard (University of Edinburgh, UK), and the cells were derived as previously described [59, 60]. The NSC were given the same media as the CSC, except they were supplemented with fibroblast growth factor (FGF; Peprotech) in addition to EGF.

### Pooled epigenetic knockout screen

GU-pBT-7 and GU-pBT-19 were transduced with a lentivirus coding for Cas9, blasticidin resistance and BFP (under an Ef1a promoter) and then selected with blasticidin and sorted with FACS for BFP expression. A guide RNA (gRNA) library with four gRNA/gene targeting 1212 genes was designed with guide sequences from the Brunello library [27] (Additional file 4: Table S4). Confirmed chromatin actors from the Epifactors Database [61] and putative chromatin actors based on protein domains [62] were selected for the library (total 1094 genes). Thirty-eight ribosomal genes were added as positive controls, 80 olfactory receptor genes and 50 non-targeting negative controls. The Cas9 cell lines were transduced with this gRNA library at MOI (multiplicity of infection) 0.3 with 1000 cells/guide in duplicates with polybrene (2 µg/ml). Cells that had taken up the lentivirus (a gRNA) were selected with puromycin two days after transduction until four days after transduction. The cells were thereafter cultured maintaining at least 500 cells/gRNA at each split and pelleted at the end point (42 days). The rank-based MaGeCK (Model-based Analysis of Genome-wide CRISPR/Cas9 Knockout) software [28] was used to analyse the next-generation sequencing data with the input library as starting reference [63]. The above experiments were performed at SciLife CRISPR Functional Genomics Unit and NGI.

### Data analysis of knockout screen and selection of hits

Based on the ranking score (lower ranking means more depleted) of the positive ribosomal gene controls, we put a hit threshold at top 250-ranking with at least three out of four concordant gRNA for both replicates. Next, all positive and negative controls were excluded and false-positive hit genes were removed based on the following criteria: (1) genes with more than 1 gRNA having multiple matches in the genome were removed as multiple cuts induce a negative growth effect on cells [64, 65] and (2) lack of RNA-seq expression under normal conditions in the GU-pBT-7 or GU-pBT-19 cell lines (Additional file 1: Fig. S7).

Next, we compared our identified hits to CRISPR screen data from more than 300 traditional cancer cell lines [25]. We excluded genes from our hits if they were a hit in more than 70% of the 300 cancer cell lines (Fig. 2A, B). This was done to filter out common essential hits.

### Ribonucleoprotein formation and individual knockout

gRNA sequences for individual validation with ribonucleoprotein (RNP; Additional file 4: Table S5) were selected from the four gRNA included in the screen library [27]. RNP was created using Alt-R® CRISPR-Cas9 crRNA XT, Alt-R® CRISPR-Cas9 tracrRNA and Alt-R® S.p. HiFi Cas9 Nuclease v3 (all from IDT, Leuven, Belgium) and prepared for nucleofection as recommended by the manufacturer (IDT). Four negative controls were included in the individual knockouts in the form of (1) non-nucleofected cells, (2) cells nucleofected with RNP containing a nonsense gRNA (non-targeting towards human genome), (3, 4) cells knocked for the olfactory receptor *OR1A2* at two different locations in the gene (Additional file 4: Table S5).

Cells were detached with Accutase, centrifuged, washed with PBS, centrifuged again and resuspended in supplemented SG Cell line solution (Lonza, Basel, Switzerland). 20 µl of this cell solution, typically containing 60 000–200 000 cells depending on the cell line, was used for one reaction and combined with 5 µl of the pre-made RNP complex and 0.5–1 µl Alt-R® Electroporation enhancer (IDT). The cell/RNP solution was nucleofected with the 4D-Nucleofector® X unit (Lonza) with an optimised pulse program for each cell line (see Fig. 3A). Warm media was added immediately after nucleofection, and the samples were incubated in 37 °C for 10 min prior to seeding in 24 well plates (Corning, New York, USA). For the validation experiments, the cells were cultured in well plates for one month and continuously imaged with the Incucyte® S3 live-cell analysis system (Sartorius, Göttingen, Germany) and counted at each split with an AO/PI (acridine orange/propidium iodide) staining (Nexcelom Bioscience, Lawrence, USA). The cells were split at near-confluence, or in a few cases after ~ 14 days if the cells did not reach near-confluence. The latter was performed for knockout conditions that were not growing well and would not reach near-confluence, in order to get a measurement of the cell growth.

### On-target and off-target effect

On-target effect of the knockout was performed ~ 10 days after knockout by ICE [34] analysis (https://ice.synthego.com). We also used this method to examine potential off-targets at two of the top putative off-target locations for the gRNA (Additional file 4: Table S6) as predicted by the Benchling software (https://www.benchling.com/) using algorithms by Hsu et al. [66].

### Immunocytochemistry

CSC were fixated with 4% paraformaldehyde, and immunocytochemistry and EdU imaging were performed as previously described [17, 67] using the following primary antibodies; SOX2 (1:1000 dilution in blocking solution, ab97959, Abcam, Cambridge, UK), GFAP (1:1000, G3893, Sigma-Aldrich, Saint Louis, USA), UBE2N (1:200, ab25885, Abcam) or CHD4 (1:250, PA5-32,181, Thermo Fisher Scientific) and secondary antibody: goat anti-rabbit or anti-mouse Alexa Fluor 488 or 594; Invitrogen. Imaging and quantification were performed with the Operetta and the accompanying Harmony software (PerkinElmer, Waltham, USA) or the Celldiscoverer 7 (imaging performed at the Centre for Cellular Imaging at the University of Gothenburg) with the accompanying Zeiss Zen Blue Software (Zeiss, Oberkochen, Germany). Quantification was performed based on fluorescent intensity to determine cells positive of a staining, and the number of positive cells was normalised against all cell nuclei (identified by DAPI staining).

### Analysis of inhibitors

Briefly, cells were seeded in 384 well plates (Corning) and a UBE2N inhibitor (NSC697923, Selleckchem, Houston, USA), dissolved in DMSO, was added to the cells one day after seeding. The cells were cultured with the inhibitor for four days and imaged with the Incucyte. At the end point, cells were analysed with the CellTiter-Glo® 2.0 cell viability assay on a Glomax Discoverer (both Promega, Madison, USA) according to the manufacturer’s instructions.

## Supplementary Information

**Additional file 1**. Supplementary figures and captions.

**Additional file 2: Supplementary table 1**. Underlying data for figure 1.

**Additional file 3: Supplementary table 2**. MaGeCK analysis of the CRISPR knockout screen.

**Additional file 4: Supplementary tables 3–6**. ED50 analysis of UBE2N inhibitor (S3), gRNA sequences used in CRISPR knockout library (S4) and for validation (S5), and PCR primers (S6).

## Data Availability

The datasets generated during the current study are available within the article and its supplementary files.

## Code accessibility

The source code of MAGeCK is freely available at https://sourceforge.net/projects/mageck/.
