# Supplementary material for: CRISPR-Cas9 knockout screen identifies novel treatment targets in childhood high-grade glioma
Source: Clin Epigenetics. 2023 May 9;15:80. doi: 10.1186/s13148-023-01498-6 (PMC10170782; doi:10.1186/s13148-023-01498-6)
Supplement: Supplementary file 1 — Additional file 1. Supplementary figures and captions. [file 13148_2023_1498_MOESM1_ESM.pdf]

Supplementary figures and legends

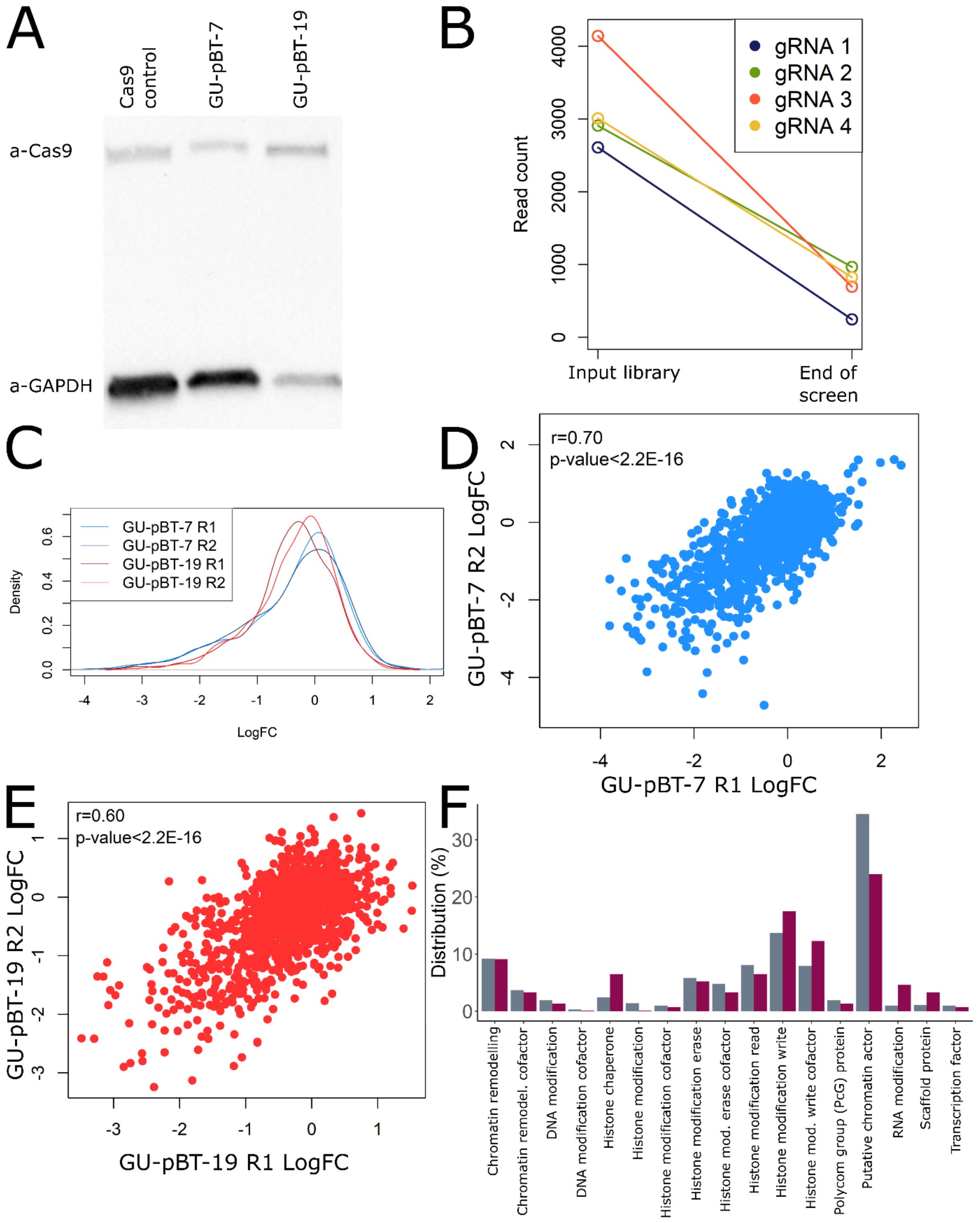

Supplementary Figure 1. A) Western blot of Cas9 verified the Cas9 expression in the transduced CSC lines. Note that the image has been cropped to include only the relevant cell lines. B) Example of a hit

gene in the screen where all four gRNA were in concordance and the gRNAs were depleted during the screen (i.e. the cells knocked for the gRNA died and/or proliferated slower). C) Density distribution of the log fold change (LFC) showed similar curves for the two technical replicates of each cell lines. The experiment was performed n=1 time. D) LFC values in replicate 1 vs replicate 2 for GU-pBT-7 and E) GU-pBT-19 respectively showed good concordance. F) Epifactor classification of the 154 hit genes in the screen.

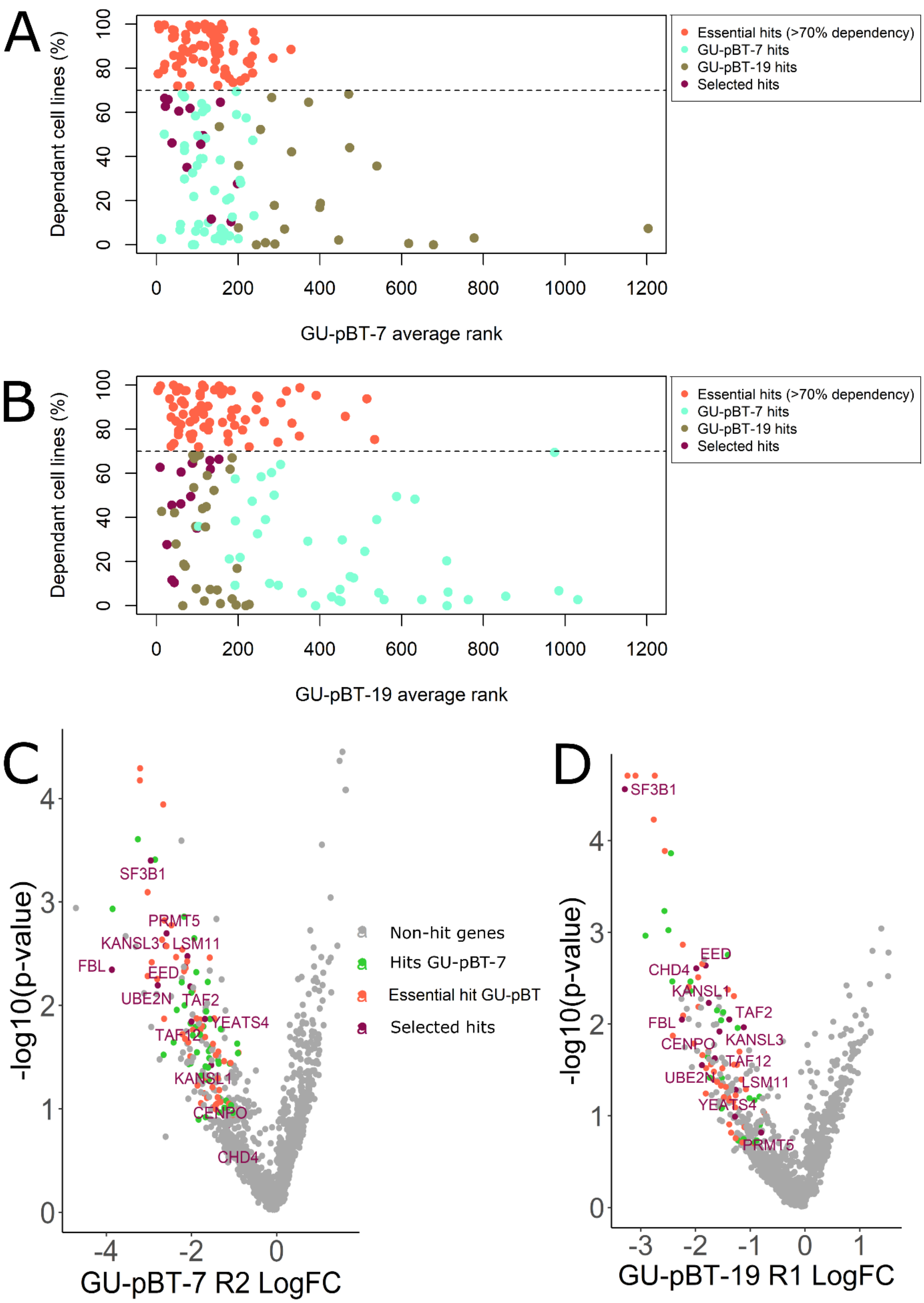

Supplementary Figure 2. A) Dependant cell lines (%) vs the average rank of n=2 technical replicates in GU-pBT-7 and B) GU-pBT-19 respectively. The experiment was performed n=1 time. Hit genes with

more than 70% dependency (dashed line) are coloured in red. C) Volcano plot for replicate 2 of GU-pBT-7 and D) replicate 1 of GU-pBT-19. The thirteen selected hits are shown in purple. Hit genes with >70% dependency is coloured in red, remaining hits in green, and remaining genes (non-hits) in grey.

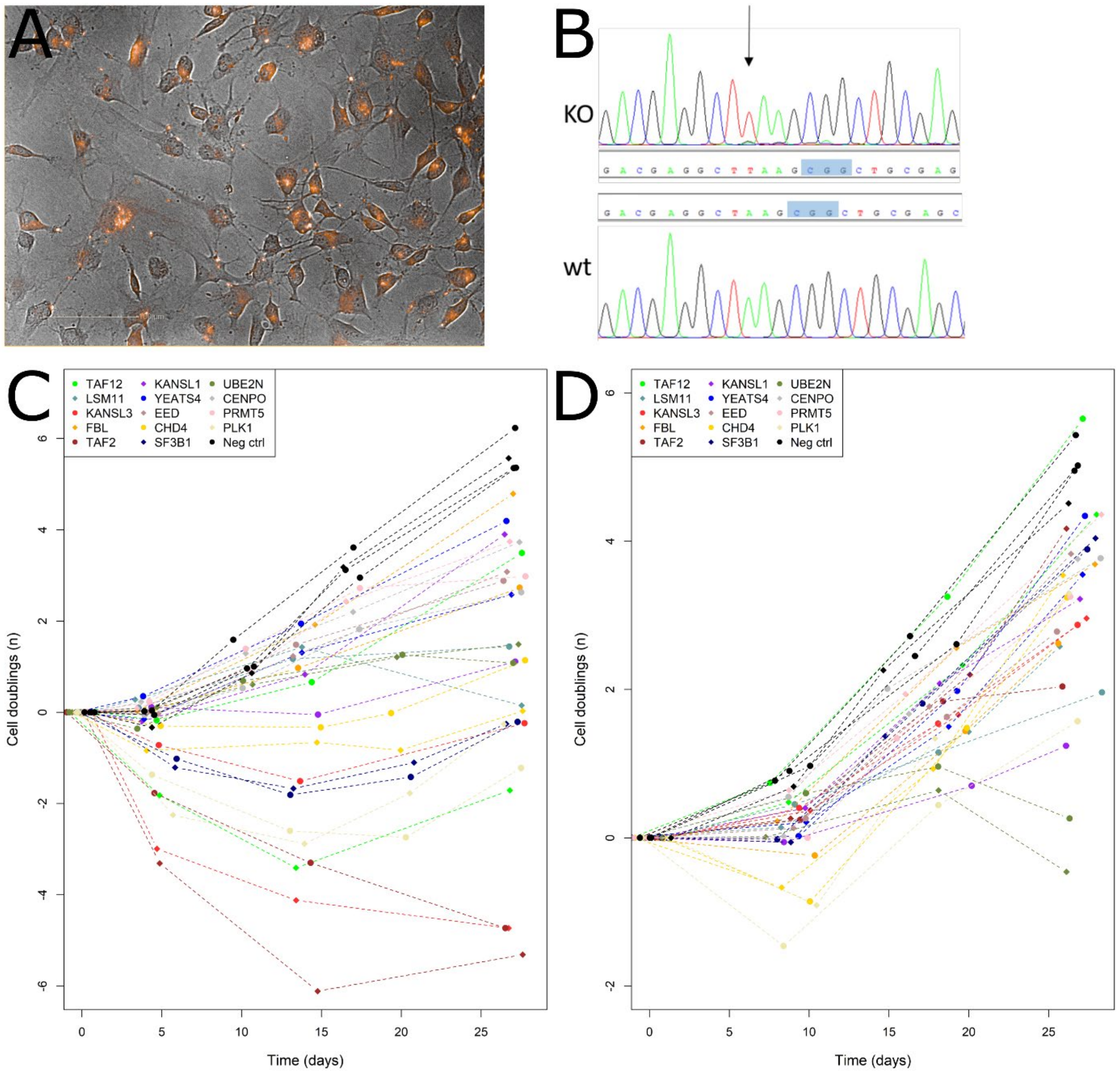

Supplementary Figure 3. A) GU-pBT-7 cells were knocked for SOX2 using a fluorescently labelled tracrRNA in the RNP complex. The successful nucleofection of the RNP complex into the cells was visualised 24 hours after transfection. B) The knockout of SOX2 (top row) led to a +1 insertion of a T (indicated by an arrow) in the target region compared to wildtype. The PAM-site is marked in blue. C) Complete cell doubling chart after knockout of the thirteen selected hits in GU-pBT-7 and D) GU-pBT-19. Note that n=two gRNA/gene (i.e. biological replicates; displayed in same colour but different shapes). The experiment was performed n=1 time.

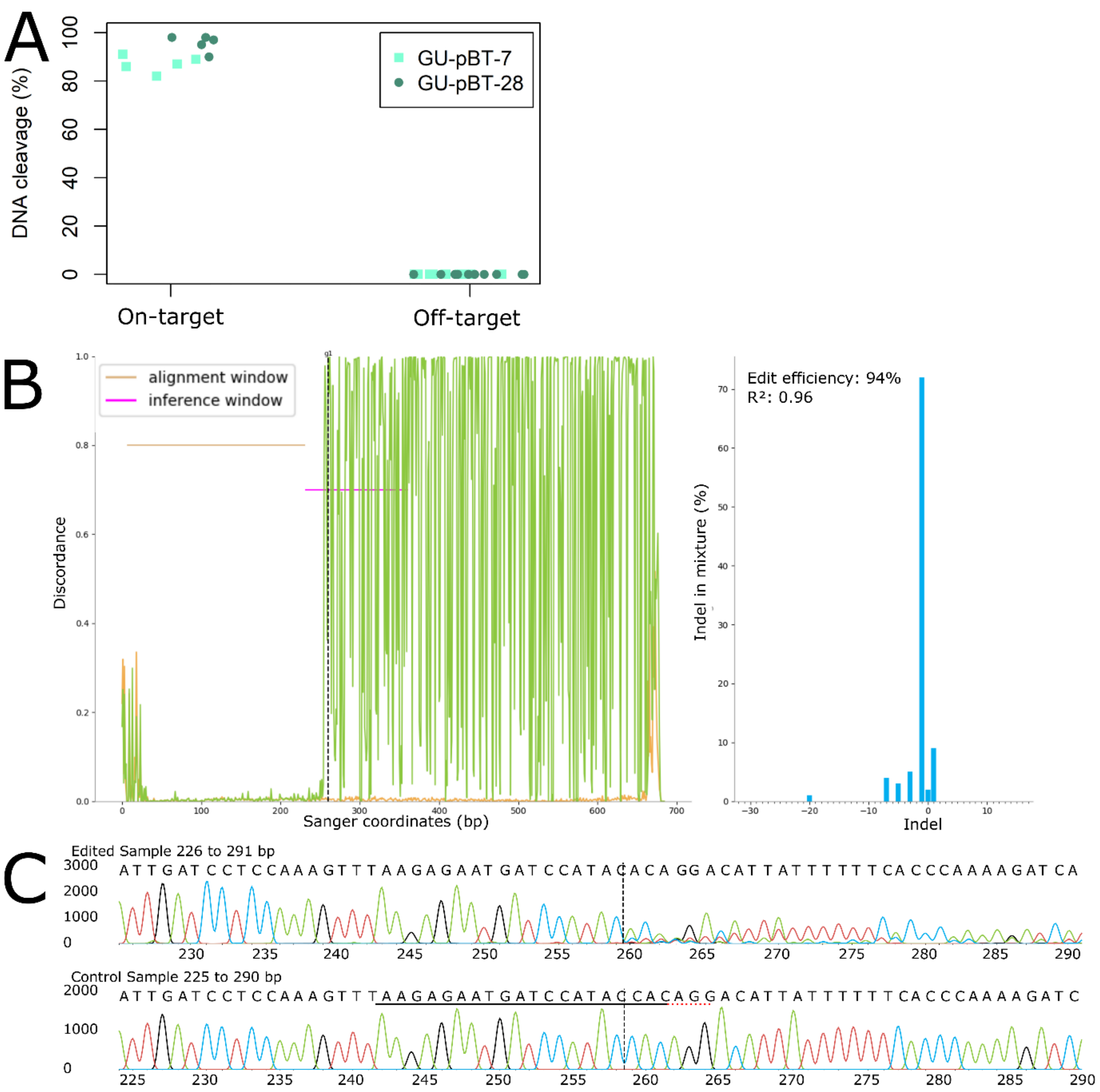

Supplementary Figure 4. A) ICE analysis showed DNA cleavage at the intended knockout site in more than 80% of the cells for the candidate genes in two CSC lines. B) Output from the ICE analysis of an EED knockout sample compared to an unknocked control with alterations occurring after the expected cut site (dashed line; left). The most common alteration was deletions of one bp and other small insertions/deletions (indel) with a total cleavage efficiency of 94% (right). C) Sanger sequencing chromatogram for the EED knockout (top) and control (bottom) shows large alterations in the knockout sample after the expected cut site (dashed line). The gRNA sequence is underlined in black and the PAM site is underlined with dashes in red.

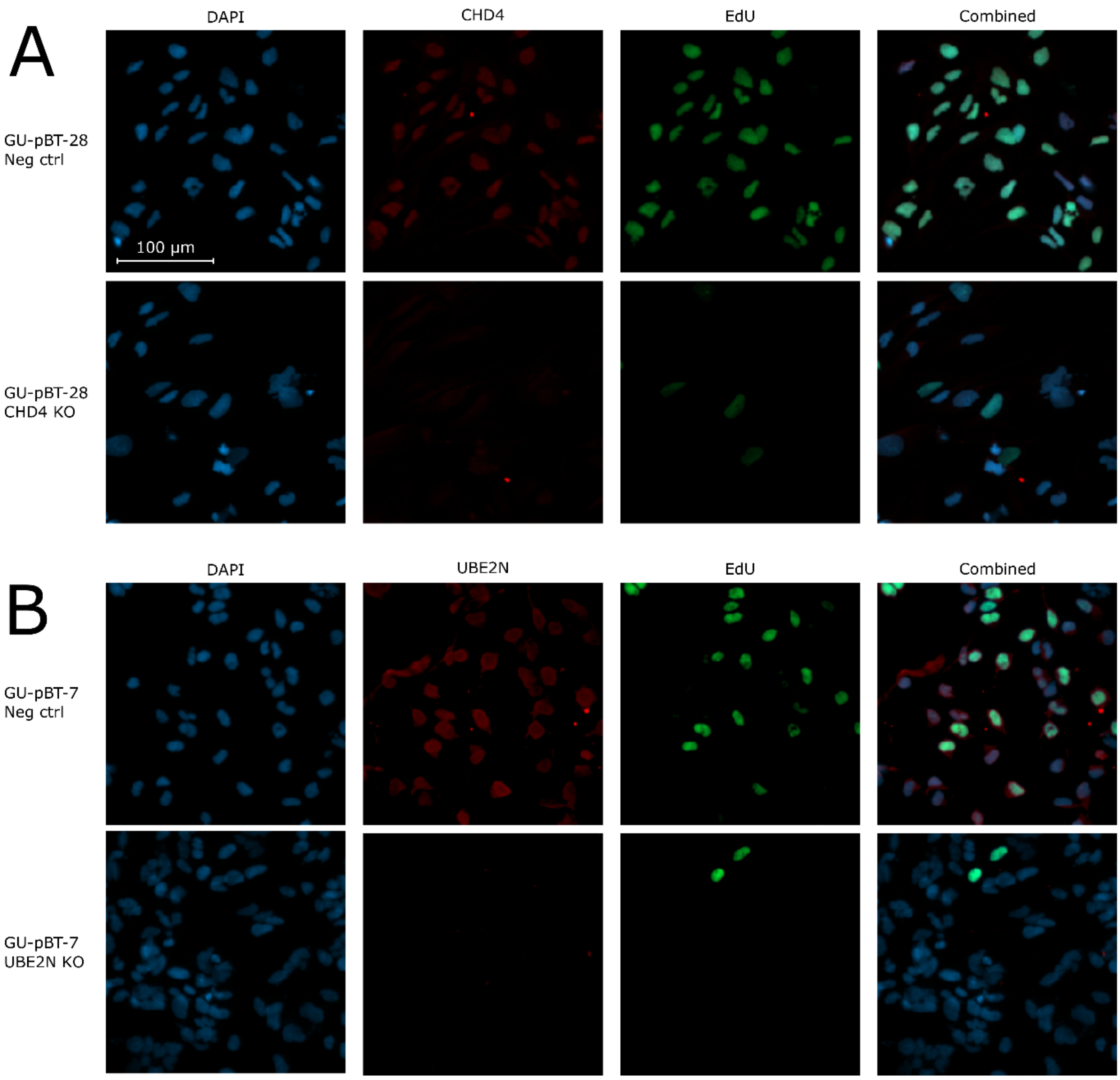

C

	CHD4+ (%)		UBE2N+ (%)	
	Control	Knockout	Control	Knockout
**GU-pBT-7**	83.28±0.62	15.57±2.65 *	95.79±0.95	2.32±1.48 *
**GU-pBT-28**	83.83±4.56	11.51±4.36 *	82.73±3.54	40.7±1.59 *
**GU-pBT-58**	92.10±0.73	17.62±5.87 *	80.01±3.63	18.49±4.04 *

Supplementary Figure 5. A) Immunocytochemistry ~10 days after knockout (bottom) demonstrates loss of CHD4 and B) UBE2N respectively compared to the negative control (top). The fluorescent channels for DAPI, CHD4/UBE2N and EdU (proliferation marker) are shown separately and combined. C) Quantification of the fraction of cells in controls and knockouts (n=2 technical replicates per condition) expressing CHD4 and UBE2N protein shows significant (p-value<0.05; Welch one-sided t-test) loss of protein in all three examined cell lines compared to the negative control.

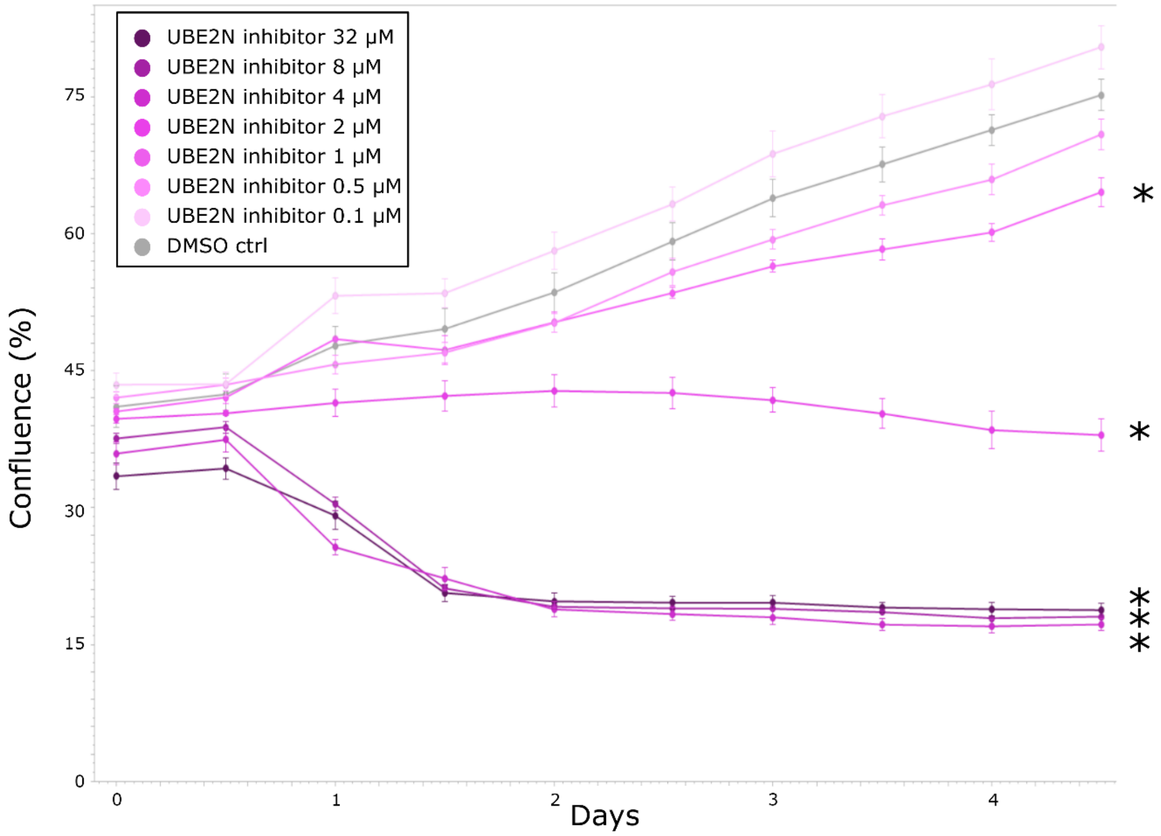

Supplementary Figure 6. The UBE2N inhibitor (NSC697923) had a rapid effect on the cells as seen by the confluence graph of GU-pBT-19 over time. The higher concentrations of the inhibitor resulted in significant growth deceleration compared to the control at endpoint (Welch one-sided t-test, p-value < 0.05; indicated with *).

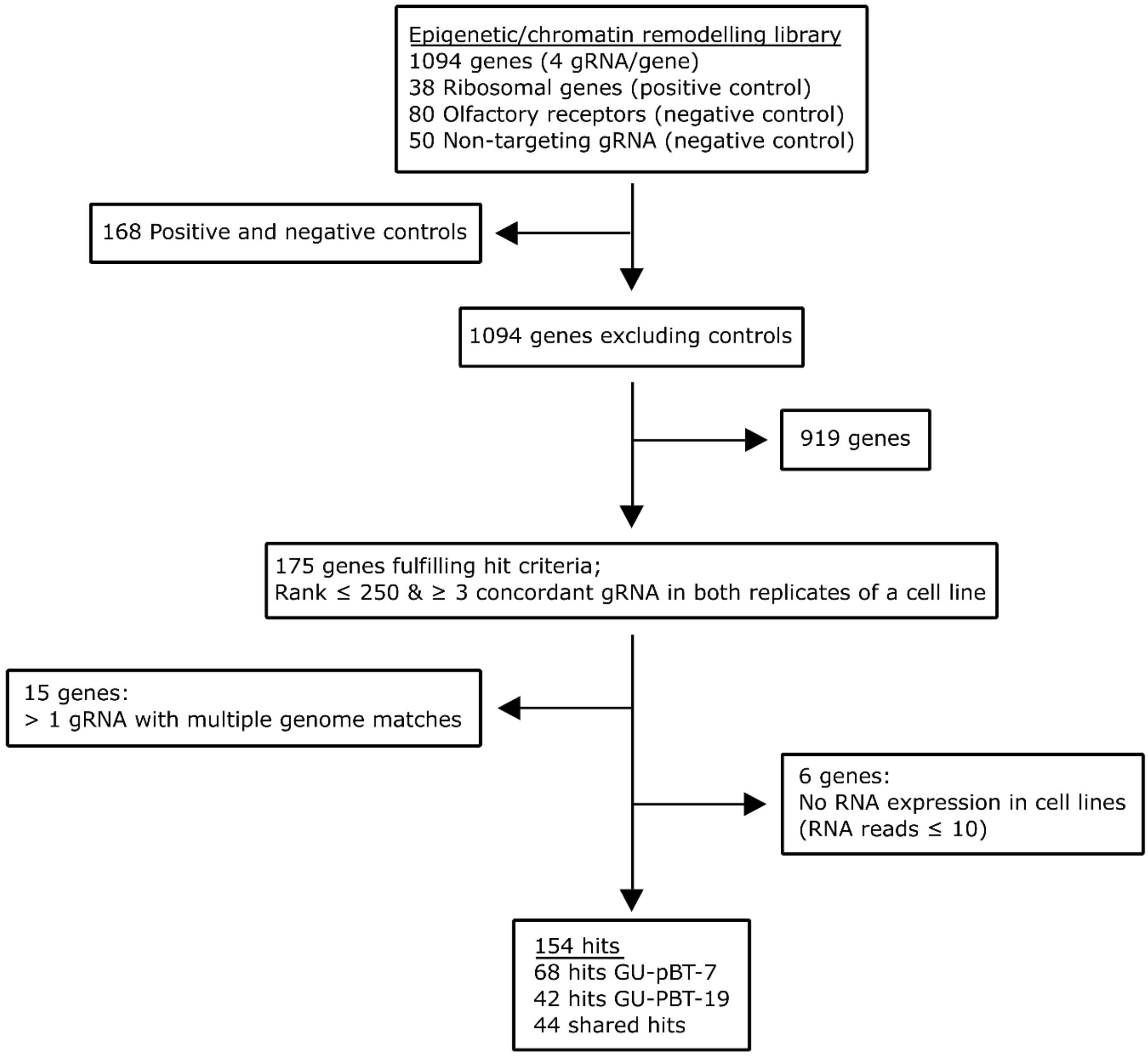

Supplementary Figure 7. Filtering strategy and thresholds for calling hits from the epigenetic/chromatin modifier knockout screen. E.g. 168 controls are filtered away, followed by 919 genes not fulfilling the hit criteria of a rank ≤250 and ≥3 concordant gRNA in both replicates of a cell line. Fifteen and six genes additionally were filtered away based on multiple matches in the genome for the gRNA sequence and lack of RNA expression in the cell lines respectively. This leaves 154 hit genes.
